# Novel Simulation Technique of Electromagnetic Wave Propagation in the Ultra High Frequency Range within Power Transformers

**DOI:** 10.3390/s18124236

**Published:** 2018-12-03

**Authors:** Takahiro Umemoto, Stefan Tenbohlen

**Affiliations:** 1 Advanced Technology R&D Center, Mitsubishi Electric Corporation, Amagasaki 6618661, Japan; 2 Institute of Power Transmission and High Voltage Technology, University of Stuttgart, 70569 Stuttgart, Germany; stefan.tenbohlen@ieh.uni-stuttgart.de

**Keywords:** power transformers, partial discharges, electromagnetic wave simulation, UHF PD measurement, UHF antennas

## Abstract

Diagnoses of power transformers by partial discharge (PD) measurement are effective to prevent dielectric failures of the apparatus. Ultra-high frequency (UHF) method has recently received attention due to its various advantages, such as the robustness against external noise and the capability of PD localization. However, electromagnetic (EM) waves radiated from PD tend to suffer attenuation before arriving at UHF sensors, because active part of the transformer disturbs the EM wave propagation. In some cases, that results in poor detection sensitivity. To understand propagation and attenuation characteristics of EM waves and to evaluate the detection sensitivity quantitatively, a computational approach to simulate the EM wave propagation is important. Although many previous researches have dealt with EM wave simulation for transformers, validations of those simulations by comparing with the experimental ones have seldom been reported. In this paper, cumulative energies, signal amplitudes and propagation times of EM waves were measured using a 630 kVA transformer. EM wave propagation was computed using the time-domain finite integration technique and the results were compared with the experimentally obtained ones. These simulation results showed good agreement with the experimental ones. The results can serve as guidelines to improve the efficiency of UHF PD detection and offer the possibility to achieve optimal placement of UHF sensors in power transformers.

## 1. Introduction

Power transformers are key components in power systems and their dielectric failures severely influence the system operation [1,2,3]. Continuous activity of partial discharge (PD), which might occur within the transformers due to undesirable local electric field enhancement, is one of the main causes of transformer failures, hence diagnoses based on PD measurement is a promising method to assess the condition of the apparatus [4].

Although various PD measurement techniques have been proposed and developed over a long period [5,6,7,8], the ultra-high frequency (UHF) method, that is, detecting electromagnetic (EM) waves in the UHF range (300 MHz–3 GHz) radiated due to a short rise time of the PD current pulse, has recently received much attention [9,10]. Attractive advantages are, for example, the robustness against external noise [11,12] and the capability of PD localization by using time-difference of arrival (TDOA) between multiple UHF sensors [13,14]. Due to these advantages, the UHF method is suitable for factory acceptance tests (FAT) and site acceptance tests (SAT), as well as on-line diagnoses [15].

However, the EM waves radiated from PD tend to suffer severe attenuation within the transformer before arriving at UHF sensors. In some cases, this results in low detection sensitivity of the PD signals, especially when the active part of the transformer (e.g., windings, core and leads) disturb the EM wave propagation [16,17]. Furthermore, localization based on TDOAs also leads to large errors due to the roundabout propagation path of the EM waves. In order to understand the propagation and attenuation characteristics of EM waves within transformers and to evaluate PD detection sensitivity as well as the propagation time quantitatively, a computational approach to simulate the EM wave propagation is essential. 

Simulation of the EM wave propagation in gas insulated switchgears (GIS) has been studied for more than 15 years and their results were compared with the theoretical or experimental ones for validation [18,19,20]. On the other hand, simulation for power transformers has also been investigated by many researchers [13,15,21,22,23]. In Reference [21], influences of transformer windings and insulation papers on amplitudes of the EM waves propagating through them were discussed based on only numerical computation. In Reference [22], the propagation times of PD induced EM signals within power transformer were computed in order to improve the accuracy of PD localization. In Reference [23], the signal amplitudes of EM waves were computed as a function of UHF sensor positions based on the simulation using an actual transformer model. However, the validity of the EM wave simulation was not discussed, hence the appropriate computational conditions were still unclarified. Considering the above fact, validations of simulations of EM wave propagation and those simulated results by comparing with the experimental ones using actual transformer structures have been seldom reported, therefore the validations are insufficient.

The objective of this paper is to propose the simulation of EM wave propagation, including the detailed simulation conditions, which are validated by the experimental results using actual transformers. First, validations of antenna modeling methods, an exciting signal as well as a model of a transformer tank were evaluated by measurement with an empty transformer tank (i.e., without active parts of a transformer). Second, cumulative energies of the EM waves, their signal amplitudes and propagation times to each UHF sensors were investigated by simulation and measurement using a distribution transformer for validating the transformer modeling. For both investigations, the simulated results showed good agreement with the measured ones. Thus, the authors successfully validated this novel simulation technique. 

The rest of this paper is organized as follows: Section 2 presents the experimental setup and measurement system of UHF signals, including a transformer structure. Detailed EM simulation methods and 3-D modeling technique are described in Section 3. In Section 4, both simulated and measured results are compared and the validity of the simulation is discussed, while conclusions and future work suggestions are presented in Section 5. 

## 2. Experimental Method

### 2.1. UHF Sensors and EM Wave Source

**Figure 1:**
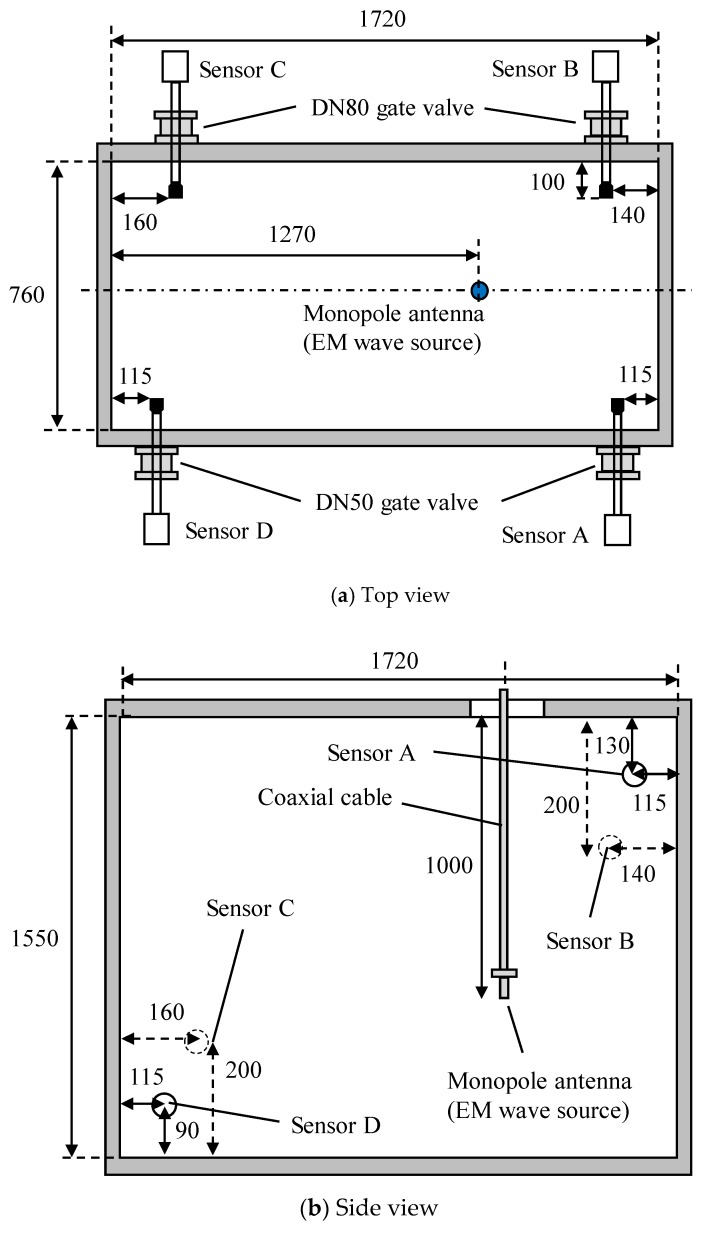
Schematic diagram of a transformer tank and antenna positions (for the first experiment).

Figure 1 illustrates a schematic diagram of a steel tank of 1350 kVA transformer and positions of four UHF drain valve sensors [11,12] and a monopole antenna in the first experiment. Inside dimension of the transformer tank was 1720 mm in length, 760 mm in width and 1550 mm in height, respectively. There was a hole with 100 mm in diameter on the top of the tank, through which a monopole antenna was inserted and used as an EM wave source. Note that in this experiment, the transformer tank was not filled with the insulating oil. 

**Figure 2:**
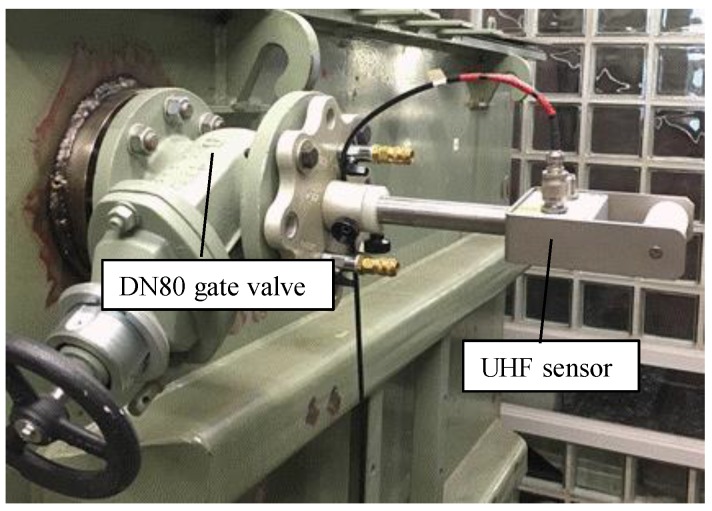
UHF drain valve sensor mounted with a DN80 gate valve.

**Figure 3:**
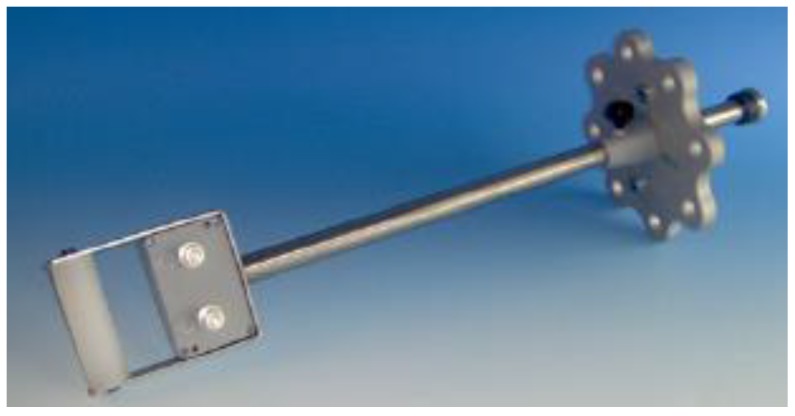
An image of the UHF drain valve sensor [4].

On the wall of the tank, there are two DN50 and two DN80 gate valves. Four UHF drain valve sensors, named A, B, C and D, were mounted with each gate valve, as shown in Figure 2. Figure 3 shows an image of the UHF sensor [4]. A probe (top of the UHF sensor) has a truncated cone shape. The detailed dimension of the probe will be described later in Section 3.2. The antenna factor (AF), which indicates sensitivity of the sensor, was described in Reference [12]. The probes of the sensors (top portion of the sensors) were inserted 100 mm into the tank, which results in a high sensor sensitivity and is suitable for the comparisons with the simulated results. EM wave signals, radiated from the monopole antenna and detected by these UHF sensors were digitized and recorded by an oscilloscope (LeCroy, WavePro 7300, Chestnut Ridge, NY, USA, 3 GHz bandwidth, 20 GS/s) without any analog filter and amplifier. 

A monopole antenna of 20 mm in length and 1.3 mm in diameter was used as an EM wave source, instead of a typical PD source (e.g., a needle-plane electrode system) since it radiates stable EM waves regarding amplitudes and frequency spectra. The antenna was excited by a voltage pulse generator (Doble Engineering, UHF calibrator LDC-7/UHF, Watertown, MA, USA) through a 50 Ω coaxial cable of approximately 2000 mm length. The output voltage of the pulse generator was set to 60 V. 

In the first experiment, time-domain signals and cumulative energies of the EM waveforms detected by the four UHF sensors were evaluated to validate the modeling of antenna, exciting signal and the transformer tank in the EM wave simulation. 

### 2.2. Active Part of the Transformer

The active part of a three-phase 630 kVA distribution transformer, which is mainly composed of high and low voltage windings, an iron core and leads, was utilized for the second experiment. Note that the transformer tank used in the experiment is larger than normally used for this active part, in order to allow the UHF sensors to be inserted deeply into the tank, resulting in improved sensitivities. 

There are four high voltage (HV) and two low voltage (LV) cylindrical windings in one phase and each HV and LV winding consists of 156 and 57 layers, respectively. However, the HV winding of one phase was removed. The innermost diameter of the LV windings, the outermost diameter of the HV windings and their height are approximately 207 mm, 350 mm and 780 mm, respectively. Three identical monopole antennas of 20 mm in length, described in Section 2.1, were also used as EM wave sources in this second experiment. These antennas were set around the windings at different heights and in different positions before the active parts were installed into the transformer tank. The detailed structure of this transformer and the positions of three monopole antennas will be illustrated in Section 3.1, as a 3-D computational model. 

**Figure 4:**
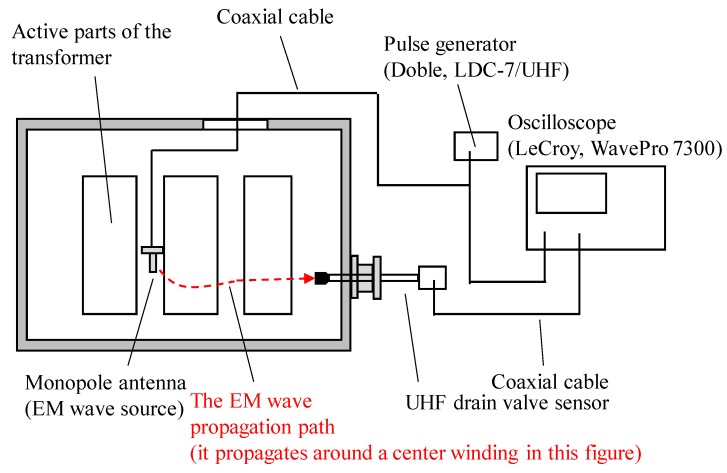
Measurement setup for propagation times of the EM waves.

In this second experiment, propagation times of the EM waves from each monopole antenna to the sensors were also measured in addition to signal amplitudes and their cumulative energies. Figure 4 illustrates the measurement setup for propagation times of the EM waves, in which the output of the pulse generator and the EM wave signals were simultaneously measured. The propagation times of the EM waves were calculated as the time difference of arrival between both signals, considering the signal propagation time within the coaxial cables. 

### 2.3. Denoising of the Cumulative Energies

Cumulative energies of the EM wave signals are commonly used not only to evaluate the PD signal strength quantitatively but also to determine the arrival times of the PD signals for the localization [21,24]. Cumulative energy *E*(*t*) of a discrete voltage waveform can normally be calculated as,

(1) $$E(t)=\sum_{i=0}^{\frac{t}{\Delta t}}\frac{V^{2}(i\cdot \Delta t)}{Z}\cdot \Delta t$$

where *V*(*i*Δ*t*) is a voltage of the EM signal at *t* = *i*Δ*t*. *Z* and Δ*t* are an impedance of the measurement circuit (usually 50 Ω) and a sampling period, respectively [21,25]. However, in this experiment, some measured signals showed low SNR (signal to noise ratio) due to the severe attenuation of the EM waves by the deflation and reflection, resulting in calculation errors of the cumulative energies.

**Figure 5:**
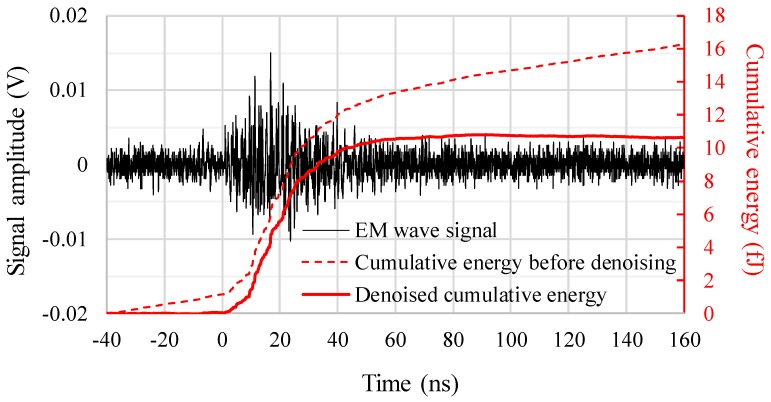
Measured EM waveform and its cumulative energy before and after denoising procedure.

In this research, in order to evaluate the cumulative energies accurately even for the low SNR waveforms, background noise components on the cumulative energies were deleted as,

(2) $$E(t)=\sum_{i=0}^{\frac{t}{\Delta t}}\frac{V^{2}(i\cdot \Delta t)}{Z}\cdot \Delta t-A\cdot t$$

where *A* is a compensation factor of the background noise, which can be obtained as a slope of the cumulative energy before the EM wave signal arrives at the sensor. Figure 5 shows an example of the measured EM waveform, its cumulative energy calculated by (1) and the denoised cumulative energy calculated by (2), respectively. Without this denoising procedure, the cumulative energy continues to increase even before the EM wave signal arrives and after it sufficiently attenuates, which leads to large errors in the total energy (i.e., the convergence energy). In a case of Figure 4, the cumulative energies at 100 ns with and without the denoising are 10.6 fJ and 16.3 fJ, respectively. The relative error is approximately 53.8%. 

## 3. Simulation Technique of the EM Wave Propagation

### 3.1. 3-D Modeling of the Transformer

The EM wave propagation within the transformer was simulated by using the CST Microwave Studio software with transient solver. The calculation in the software is based on the finite integration theory (FIT), in which the Maxell’s equations are numerically solved, not in differential forms used in the finite-difference time-domain (FDTD) method [26] but integral forms [23,27]. 

**Figure 6:**
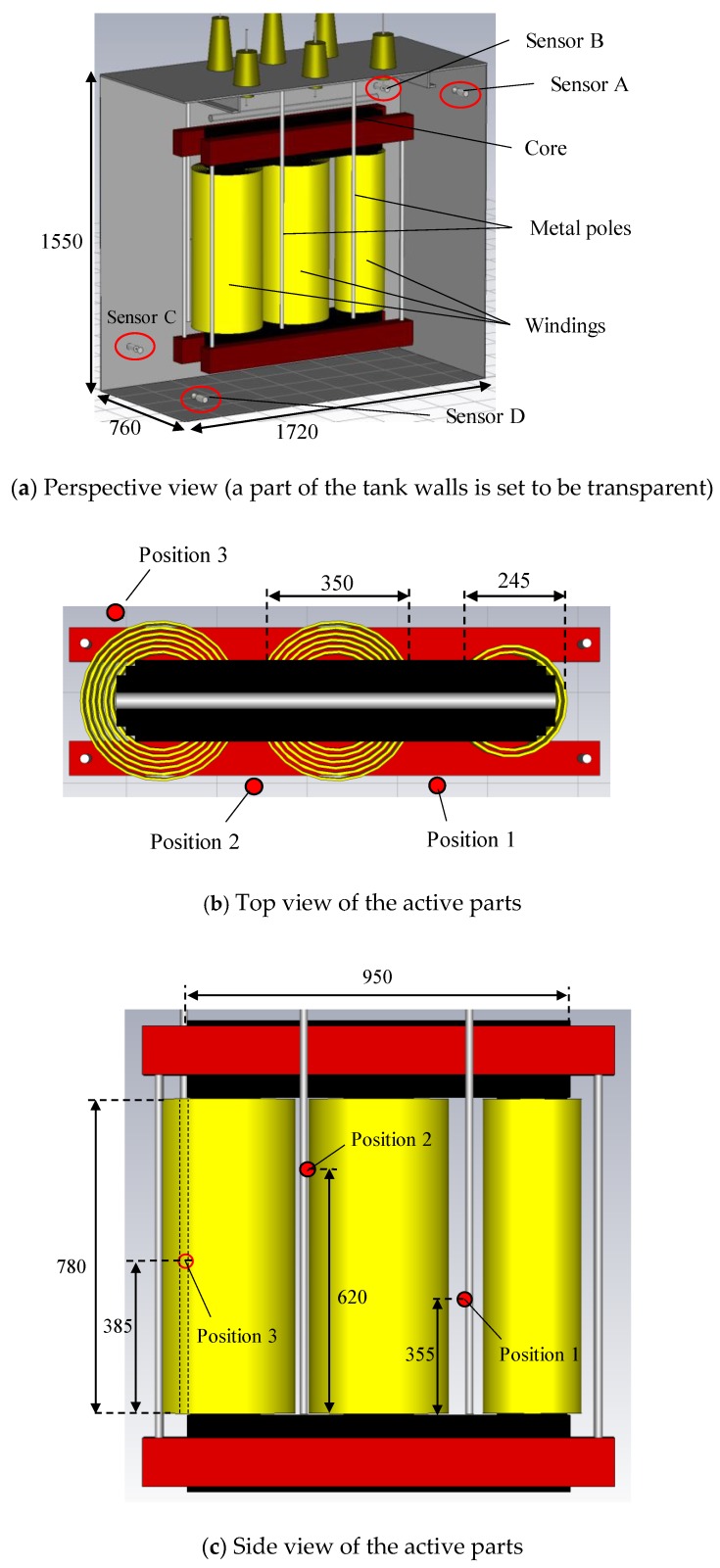
3-D computational model of the transformer and positions of the three monopole antennas.

Figure 6 shows a 3-D computational model, which simulates the transformer tank and the active part of the 630 kVA transformer, which was used in the experiment as illustrated in Figure 1. Basically, the structure of the active part and its size in this model are the same as the actual ones. However, HV and LV leads, which connect both windings to bushings on the top of the tank, were not modeled due to their complicated structures. Furthermore, each winding was modeled as a conductive cylinder to make the model simple and reduce the computational time drastically. This simplification is possible, because the windings of this transformer used in this study are the cylindrical type, hence there is no oil gap between each layer. In fact, there are quite small gaps between conductors because of the layer-insulation and the EM waves can theoretically propagate through them to some extent. However, these propagating paths can be ignored in this research because the EM waves attenuate severely and cannot be detected experimentally. The validations of this transformer modeling will be discussed in Section 4.2.

The positions of the three monopole antennas (EM wave sources) are also indicated in Figure 6. Two of them, named positions 1 and 2, are located at the same side around the center windings but at different heights. Another EM source, position 3, is at the other side of the windings. 

### 3.2. Antenna Modeling

A feeding method of an antenna for the simulation has not been established yet, although several methods have been proposed [28,29,30,31,32]. In this paper, the gap feeding method [33,34] was applied to simulate both the monopole antenna for radiating the EM waves and the UHF sensors for receiving them, due to its simplicity. In this feeding method, a coaxial cable to feed the antenna was not modeled, while the feeding port (e.g., voltage source) was introduced at the gap between a probe (e.g., monopole) and grounded conductor. 

**Figure 7:**
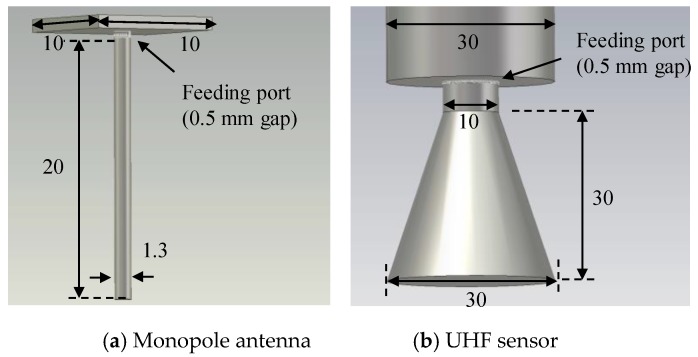
Modeling of the monopole and UHF sensors.

Figure 7 illustrates models of the monopole and UHF antennas with the gap feeding. The monopole in the simulation model was 20 mm in length and 1.3 mm in diameter with a plate conductor having an area of 10 mm × 10 mm. The probe of the UHF sensor was a circular truncated cone shape with a bottom diameter of 30 mm, a top diameter of 10 mm and a height of 30 mm and it had a conductor with a diameter of 30 mm. The gap lengths of the feeding port were set to 0.5 mm for both the monopole and UHF sensors and their impedance was set to 50 Ω. An exciting voltage signal was applied to the feeding port of the monopole antenna and the received voltage waveform across the port of the UHF sensors were analyzed. 

### 3.3. Other Computational Conditions

Table 1 presents conductivity, relative permittivity and permeability of the materials used in the EM wave simulation. On the surface of the copper of the transformer windings, oil-impregnated paper 0.3 mm thick was set as a coating material to represent the layer insulation of the windings. 

When a voltage pulse is applied to an antenna, the voltage waveform at the antenna terminal is determined both by the frequency-dependent input impedance of the antenna and characteristic impedance of the coaxial cable [35]. Generally, it is not easy to determine the actual exciting signal of the antenna. In this research, the exciting voltage waveform was determined based on the reflected voltage waveform from the open-ended top of the antenna, as proposed in Reference [25]. The amplitude and rise time (10–90%) of the exciting signal applied for the simulation were set to 60 V and 0.8 ns, respectively. 

The hexahedral mesh was used in this computation. The frequency range and cell numbers per wavelength were set to 0–1500 MHz and 30, respectively. This results in approximately 76,000,000 total mesh cells. The time durations of the simulation were set to 800 ns for the first experiment without the active parts and 100 ns for the second experiment. At these times, the EM waves, propagating within the tank, attenuated sufficiently to evaluate the convergence cumulative energies. 

## 4. Evaluations of the Simulated Results and Discussions

### 4.1. Validations of the Antenna and Transformer Tank Modeling

For the first step, the EM wave propagation in the tank without the active parts of the transformer was simulated and the results were compared with the measured ones in order to remove the influence of the active parts and validate the modeling technique of the monopole and UHF sensors as well as the transformer tank.

**Figure 8:**
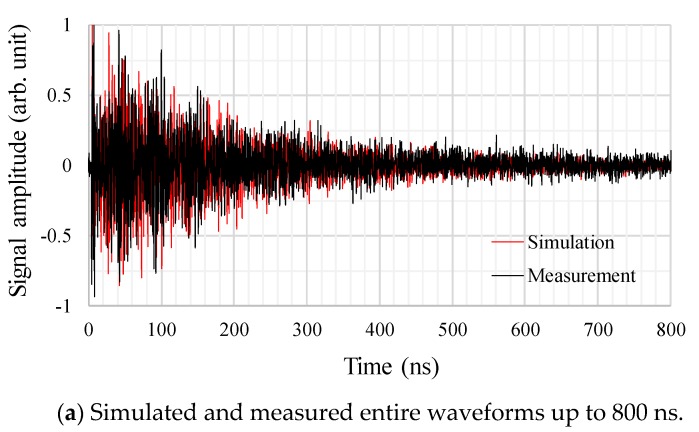

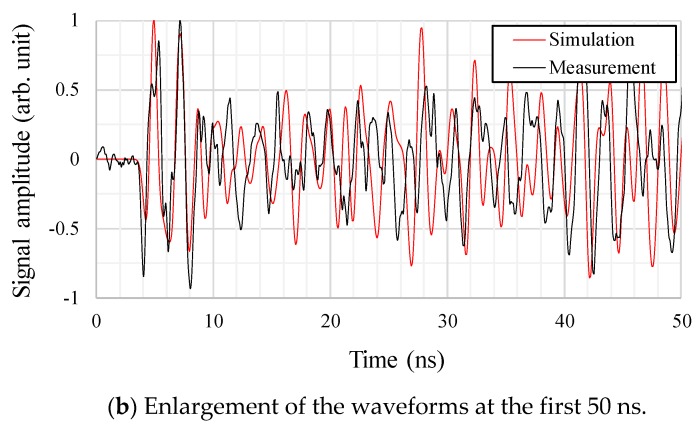
Examples of the simulated and measured EM waveforms by sensor A.

Figure 8 shows an example of the simulated and measured EM waveforms by sensor A. In this figure, the signal amplitudes of both the simulated and measured values were normalized by the maximum signal strength of each waveform. Figure 8a,b show the entire waveforms up to 800 ns and the enlargements of the first 50 ns, respectively. From these figures, it can be seen that the attenuation degrees of the signal amplitudes and the time-domain EM waveforms by the simulation and measurement showed quite good agreement with each other, especially for the first 10 ns in Figure 8b. Such a good agreement of the simulated waveform with the measured one has never been reported before.

**Figure 9:**
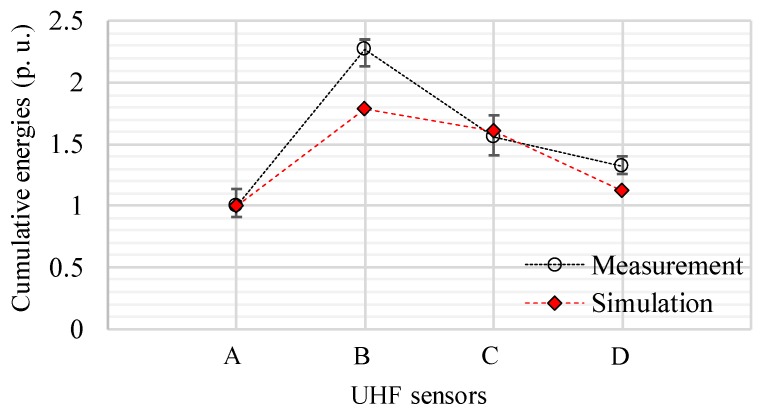
Simulated and measured cumulative energies as a function of the UHF sensor positions.

**Figure 10:**
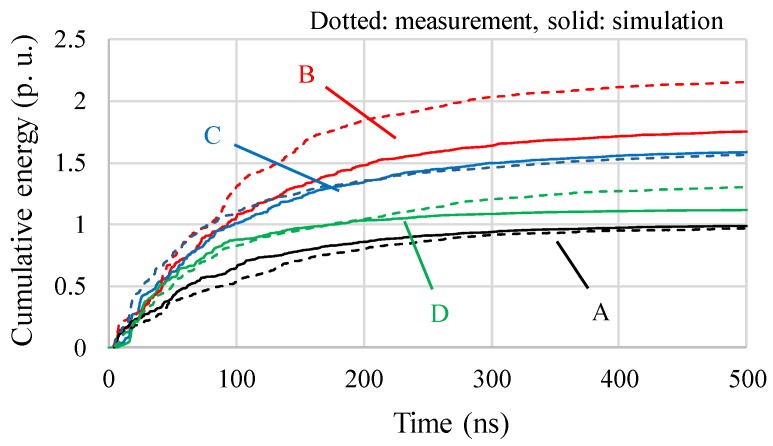
Cumulative energies as a function of time for the four UHF sensors.

Figure 9 compares the simulated and measured cumulative energies for the four UHF sensors. In this figure, the cumulative energies were normalized by the values by sensor A. Both the simulated and measured results showed a similar trend that the sensors A and B showed the lowest and highest sensitivities, respectively, although the maximum error between the simulated and measured results was about 21% for sensor B. Figure 10 shows cumulative energies as a function of time for the four UHF sensors. Although convergence values of the cumulative energies by the simulation and measurement showed some differences, especially for sensor B as expected from Figure 9, the degree of increase in the cumulative energies in the simulation showed reasonable agreement with the measured ones. 

Based on the results of the first experiment, presented in Figure 8, Figure 9 and Figure 10, it can be said that the modeling techniques of the monopole and the UHF sensors, the exciting voltage waveform as well as the tank modeling, including the material properties, are reasonable. 

### 4.2. Propagation Times of the EM Waves within a Transformer 

As the next step, the active parts of the transformer were installed into the tank. In order to validate the size and positions of the transformer winding model in the simulation, propagation times of the EM waves from the EM wave sources to each sensor were computed and compared with the experimental and theoretically calculated ones. In the experiment, the propagation times were obtained by simultaneous measurement of the exciting signal and the resultant EM wave signals, where arrival times of the EM waves were systematically calculated based on the Energy criterion method [8,21]. 

**Figure 11:**
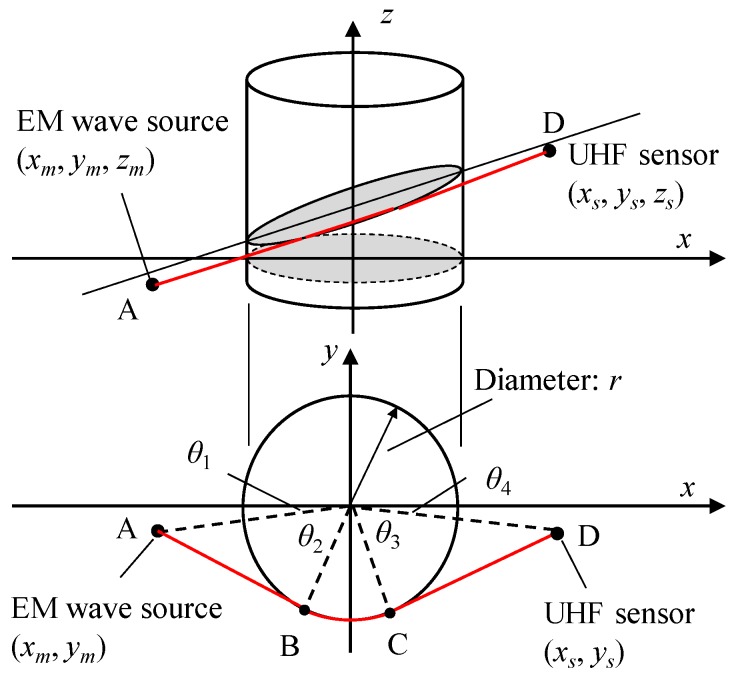
Propagation path of the EM waves around a cylindrical obstacle and its 2-D projection.

Theoretical propagation times were calculated, assuming the transformer windings as simple cylindrical obstacles [36]. Figure 11 illustrates geometrical model of the propagation path around a cylinder and its 2-D projection. In a 2-D projection, we assume coordinates of the EM wave source and UHF sensor as (*x_m_*, *y_m_*) and (*x_s_*, *y_s_*). Then, *θ*_1_, *θ*_2_, *θ*_3_ and *θ*_4_ illustrated in Figure 11 can be expressed as,

(3) $$\theta_{1}=\tan^{-1}(y_{m}/x_{m})$$

(4) $$\theta_{2}=\tan^{-1}(\sqrt{x_{m}{}^{2}+y_{m}{}^{2}-r^{2}}/r)$$

(5) $$\theta_{3}=\tan^{-1}(\sqrt{x_{s}{}^{2}+y_{s}{}^{2}-r^{2}}/r)$$

(6) $$\theta_{4}=\tan^{-1}(y_{s}/x_{s})$$

where *r* is a radius of the cylindrical obstacle. Considering the height difference in 3-D, the propagation distance from the source to the sensor, *L_AD_*, is expressed as,

(7) $$\begin{array}{l} L_{AD}={\{[\left(x_{m}{}^{2}+y_{m}{}^{2}-r^{2}\right)}^{1/2}+r\cdot (\pi -\theta_{1}-\theta_{2}-\theta_{3}-\theta_{4})\\ +{\left(x_{s}{}^{2}+y_{s}{}^{2}-r^{2}\right)}^{1/2}]+{{\left(z_{m}-z_{s}\right)}^{2}\}}^{1/2} \end{array}$$

where *z_m_* and *z_s_* denote *z* coordinates of the EM wave source and the sensor, respectively [36]. Theoretical propagation time considering the obstacles can be calculated by *L_AD_*/*c*, where *c* is the light speed in this case.

**Figure 12:**
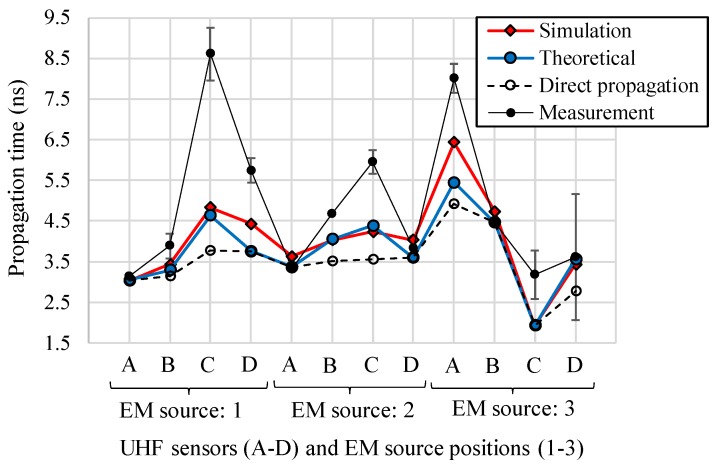
Propagation times of the EM waves by the simulation, experiment and theoretical calculations.

Figure 12 shows propagation times of the EM waves, obtained by the simulation, the experiment and theoretical calculation described above as functions of positions of the UHF sensors and the EM wave sources. For comparison, direct propagation times were also plotted, which were calculated based on the Euclidian distance between an EM source and a UHF sensor divided by the light speed. It can be seen that the simulated and theoretically calculated propagation times showed quite good agreement for all UHF sensors and the source positions, while in some cases, there are some differences of about 1 ns between the simulated results and those calculated assuming direct propagation. This fact indicates that the influences of the active part on the propagation times were accurately simulated and thus the modeling of the transformer active part, mainly the positions and size of the windings, was successfully validated. 

It should be noted that some measurement results (e.g., sensor C in EM source position 1 or sensor A in EM source 3) showed large errors from the other results. These were caused by the roundabout propagation path and resulting severe attenuations of the EM waves, which made the arrival of the EM signals unclear. These large errors in the determination of the arrival time will lead to a critical PD localization error, so the influence on the location accuracy will be evaluated in future works. 

### 4.3. Cumulative Energies and Signal Amplitudes as a Function of Sensor Positions 

Finally, cumulative energies and signal amplitudes of the EM wave signals from the simulations were evaluated by comparing with those from the experimental using the active parts of the transformer in order to validate the newly developed simulation technique. 

**Figure 13:**
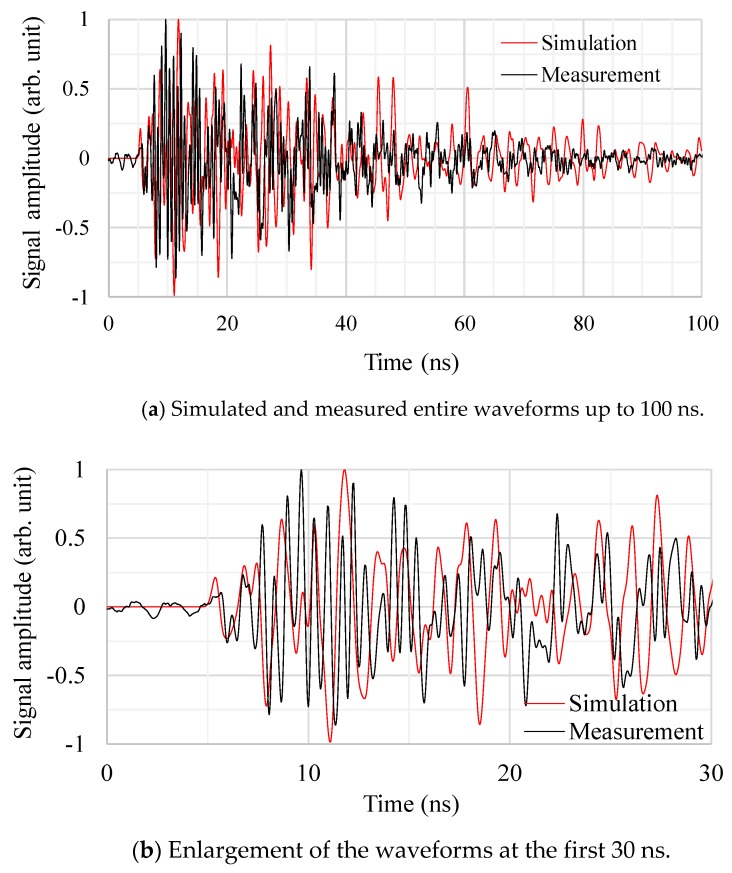
Example of the simulated and measured EM waveforms. (EM wave source position 3, UHF sensor C).

Figure 13 shows the entire and the first 30 ns of the time-domain EM waveforms, which were obtained by sensor C at the EM wave source position 3, respectively. The signal amplitudes of both the simulated and measured values were normalized by the maximum signal strength of each waveform. From Figure 13a, the attenuation degree of the EM waves as a function of time agreed well with the measured one. Furthermore, the EM waves attenuated sufficiently within 100 ns, while in the first experiment without the active parts, it took more than 500 ns as shown in Figure 8a. This rapid attenuation was caused by increasing the reflection and diffraction of the EM waves due to the active parts. Also, Figure 13b indicates that the waveforms both by the simulation and measurement were quite similar up to 50 ns. These agreements in Figure 13a,b suggest that the modeling of the active part of the transformer is reasonable. 

**Figure 14:**
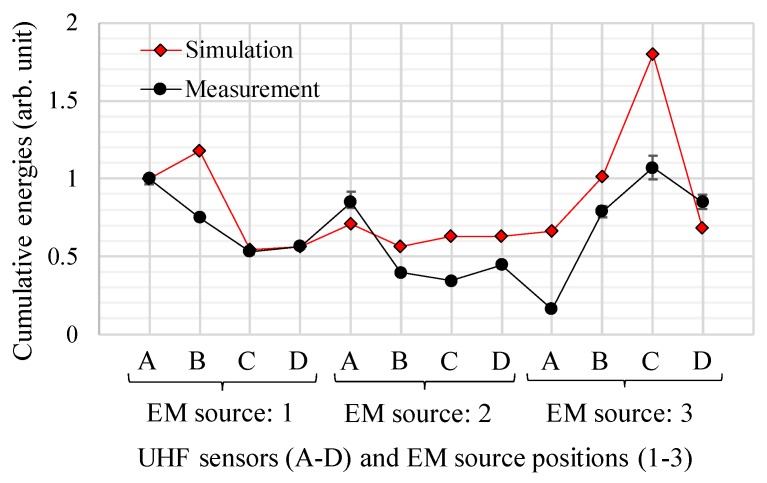
Simulated and measured cumulative energies for each UHF sensors and EM wave source.

**Figure 15:**
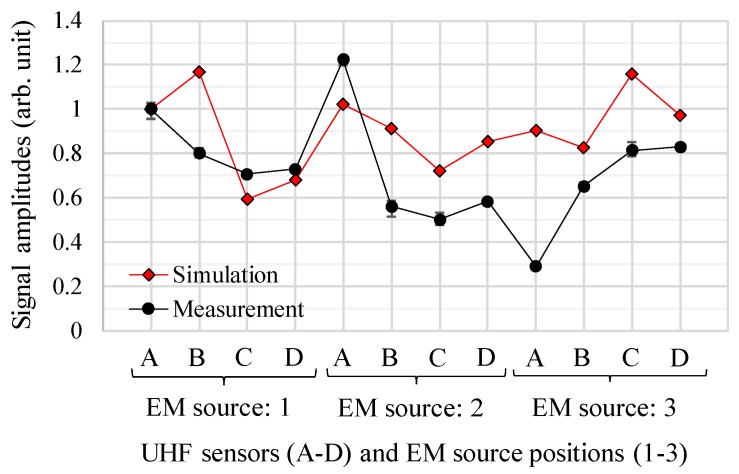
Simulated and measured signal amplitudes for each UHF sensors and EM wave source.

Figure 14 and Figure 15 show the simulated and measured cumulative energies and signal amplitudes as functions of the source and the sensor positions, respectively. In both figures, the vertical axes were normalized by the values from sensor A at the source position 1. For both cumulative energies and signal amplitudes, on the whole, the simulated results show a similar trend to the measured results. However, the cumulative energies show better agreement, because the signal amplitudes tend to be strongly affected by resonances of the EM waves, which are difficult to simulate accurately. 

As shown in Figure 12, Figure 13, Figure 14 and Figure 15, the propagation times of the EM waves, the time-domain EM waveform and signal strength (i.e., cumulative energies and their amplitudes) as a function of the sensor position by the simulation showed reasonable agreement with the measured ones. Thus, the newly developed simulation technique for the EM wave propagation has been successfully validated. Furthermore, it firstly enables us to investigate the sensitivities of PD measurement as a function of UHF sensor positions for actual transformers by computation.

The authors believe that this simulation technique will contribute to further investigations for the optimization of the UHF sensor positions, their numbers as well as the type of sensor, by applying the antenna modeling technique described in Section 3.2 and the sensitivity investigation as a function of sensor positions in Section 4.3.

## 5. Conclusions

The authors have proposed a simulation technique for EM wave propagation within transformers and validated the simulated results by comparing with those experimentally obtained, using a 630 kVA distribution transformer. 

First, validities of modeling methods for a monopole antenna as an EM wave source, UHF sensors as well as the transformer tank were investigated by comparing with experimental results obtained with an empty transformer tank. Consequently, the simulated time-domain EM waveforms, the attenuation rate of EM wave strengths and cumulative energies as a function of UHF sensor position showed good agreement with the measured ones. Therefore, those modeling methods were successfully validated.

Second, propagation times, signal amplitudes and cumulative energies of the EM waves were evaluated by simulation, measurement and theoretical consideration by using a 630 kVA distribution transformer in order to confirm the validation of the modeling of the active parts of a transformer. As a result, the simulated EM waveforms, their propagation times, cumulative energies and signal amplitudes as a function of UHF sensor position showed reasonable agreement with the experimentally and theoretically obtained ones. This suggests that the computational conditions, including the modeling of the transformer structure were appropriate.

Based on these results, this newly developed simulation technique, proposed in this paper, will contribute to the optimization of the UHF sensor positions and their numbers as well as the type of UHF sensors to obtain the desired PD detection sensitivity for power transformers. 

## Figures and Tables

**Table 1:** Material properties used in the simulation.

Material (Model Component)	Conductivity (S/m)	Relative Permittivity	Relative Permeability
Air	0	1	1
Copper (winding model)	6.0 × 10^7^	∞	1
Paper (layer-insulation)	1.0 × 10^−14^	3.9	1
Silicon steel (core)	1.0 × 10^5^	∞	6000
Steel (tank)	5.0 × 10^5^	∞	500
